# Progressive auto-segmentation for cone-beam computed tomography-based online adaptive radiotherapy

**DOI:** 10.1016/j.phro.2024.100610

**Published:** 2024-07-14

**Authors:** Hengrui Zhao, Xiao Liang, Boyu Meng, Michael Dohopolski, Byongsu Choi, Bin Cai, Mu-Han Lin, Ti Bai, Dan Nguyen, Steve Jiang

**Affiliations:** Medical Artificial Intelligence and Automation Laboratory and Department of Radiation Oncology, University of Texas Southwestern Medical Center, Dallas, TX 75390, USA

**Keywords:** RNN, LSTM, Adaptive radiotherapy, Segmentation, CBCT, Prior knowledge

## Abstract

**Background and purpose:**

Accurate and automated segmentation of targets and organs-at-risk (OARs) is crucial for the successful clinical application of online adaptive radiotherapy (ART). Current methods for cone-beam computed tomography (CBCT) auto-segmentation face challenges, resulting in segmentations often failing to reach clinical acceptability. Current approaches for CBCT auto-segmentation overlook the wealth of information available from initial planning and prior adaptive fractions that could enhance segmentation precision.

**Materials and methods:**

We introduce a novel framework that incorporates data from a patient’s initial plan and previous adaptive fractions, harnessing this additional temporal context to significantly refine the segmentation accuracy for the current fraction’s CBCT images. We present LSTM-UNet, an innovative architecture that integrates Long Short-Term Memory (LSTM) units into the skip connections of the traditional U-Net framework to retain information from previous fractions. The models underwent initial pre-training with simulated data followed by fine-tuning on a clinical dataset.

**Results:**

Our proposed model’s segmentation predictions yield an average Dice similarity coefficient of 79% from 8 Head & Neck organs and targets, compared to 52% from a baseline model without prior knowledge and 78% from a baseline model with prior knowledge but no memory.

**Conclusions:**

Our proposed model excels beyond baseline segmentation frameworks by effectively utilizing information from prior fractions, thus reducing the effort of clinicians to revise the auto-segmentation results. Moreover, it works together with registration-based methods that offer better prior knowledge. Our model holds promise for integration into the online ART workflow, offering precise segmentation capabilities on synthetic CT images.

## 1 Introduction

The advanced techniques of radiation therapy allow us to deliver the prescribed dose to the target while minimizing dose to the surrounding normal tissues. However, anatomical changes due to tumor shrinkage, patient weight loss, or other anatomical changes during the treatment course may degrade the plan quality which is prepared before treatment [1]. Adaptive radiation therapy (ART) rectifies this issue by modifying treatment plans based on updated imaging, ensuring that the therapy is tailored dynamically throughout the course of treatment rather than remaining fixed [2]. The adapted treatment plan can further lower the dose to normal tissue while maintaining coverage to the target [3], [4], [5], [6], [7], [8], [9]. In cone-beam computed tomography (CBCT) based ART, the CBCT acquired before each fraction provides the main source of information about anatomical changes for plan adaptation.

ART can be performed offline or online [10], [11], [12]. In online ART, the daily treatment plan is adapted to the patient’s changing anatomy based on their daily CBCT image acquisition. The online nature of the treatment demands high efficiency since the patient stays on the treatment couch while waiting for treatment to start. The time-consuming process of segmenting the tumor volumes and organs-at-risk (OARs) has become a major bottleneck for the widespread use of online ART, warranting an urgent need for accurate auto-segmentation tools [13]. Auto-segmentation on CBCT is a challenging task primarily because CBCT images tend to have more noise, more artifacts, and less soft tissue contrast than computed tomography (CT) images [14]. Therefore, expert clinicians must retrospectively contour large sets of CBCT images specifically for CBCT segmentation research, which is time-consuming and leads to poor auto-segmentation results on CBCT images [15], [16], [17], [18], [19].

The current methods for auto-segmentation for CBCT-based ART can be categorized into three classes: 1. deformable image registration (DIR)-based auto-segmentation; 2. deep learning (DL)-based direct auto-segmentation; 3. hybrid auto-segmentation. DIR-based auto-segmentation methods, which are the most popular ones used in ART clinical workflows, require deforming the planning CT (pCT) to the CBCT’s anatomy and propagate contours from the pCT to the CBCT [18], [20], [21]. DL-based direct segmentation (DS) methods take the segmentation task as a classification problem by utilizing convolutional-style networks to identify objects in the image [15], [17], [19], [22]. Despite the advancement of auto-segmentation techniques [18], [23], [24], the segmentation accuracy is still far from acceptable clinical standards. Hybrid approaches by combining DIR and DS for CBCT auto-segmentation were proposed by several groups and one of the combination methods has been used in clinical ART systems. Besides, some groups proposed to get propagated contours first by DIR, followed by registration-guided DS taking both CBCT image and deformed pCT contours as input [16], [17], [21], [25]. Those studies showed that DIR and DS combined models performed better than DIR-only or DS-only models [16], [21], [25].

While existing studies have leveraged knowledge from pCT to enhance segmentation accuracy, they consistently overlook the valuable insights that could be drawn from previous treatment fractions. This oversight results in models repeatedly committing the same errors in each fraction, as they lack the capacity to retain and learn from past inaccuracies. Consequently, clinicians are tasked with correcting identical mistakes made by these models, an inefficiency that occurs at every fraction.

To address the aforementioned challenge, we approach CBCT-based auto-segmentation as a progressive task. Our segmentation model is designed to incrementally learn from the label of each fraction, while also retaining memory from prior fractions to inform the current segmentation. We have developed LSTM-UNet, an enhanced 3D U-Net-based model augmented with Long Short-Term Memory (LSTM) modules, designed to harness comprehensive prior information from pCT and earlier treatment fractions. Unlike typical applications of LSTM units in processing time-series texts, we used 3D convolutional LSTM units which adapt at maintaining a multi-scale memory of the patient's previous contoured images and predict the contours of latest images. Utilizing this retained historical data, the model is equipped to deliver more accurate segmentation predictions for the current fraction. Our method can leverage the contours of registration-based methods such as PACS [26] and TTO [20] as input and further improve the accuracy.

In this study, our contributions are as follows:

1. We introduce an innovative auto-segmentation model tailored for CBCT-based online ART that incrementally learns from each successive fraction, incorporating cumulative knowledge from all prior fractions.
2. We construct a specialized 3D LSTM-UNet, integrating LSTM modules, which effectively retain learned information across previous fractions to enhance the segmentation precision of the current fraction.
3. We conduct comprehensive experiments to benchmark the performance of our proposed model against other segmentation models that either do not incorporate prior knowledge or lack an embedded memory mechanism. Furthermore, we compare our model's efficacy with a state-of-the-art DIR-based segmentation model.

## 2 Materials and methods

### 2.1 Prior-guided auto-segmentation in CBCT-based online ART

In a typical online ART system, pCT images undergo transformation to create a synthetic CT (sCT), crucial for precise dose calculations. Notably, the existing workflow predominantly relies on the baseline plan—usually the initial pCT and its associated plan established before the first fraction—and overlooks valuable insights from subsequent fractions that could inform the segmentation for the current day.

**Fig. 1:**
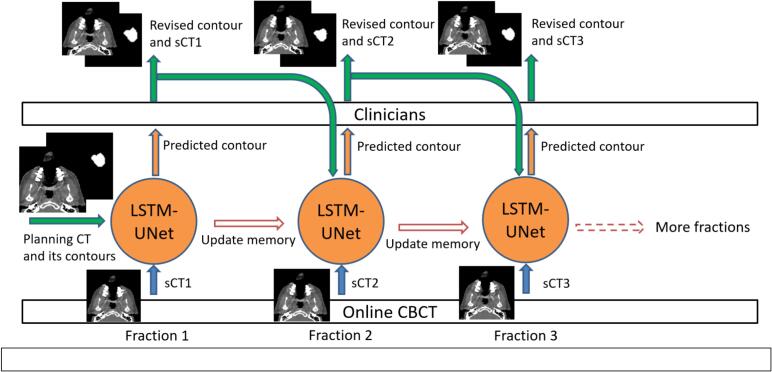
The proposed workflow of auto-segmentation for CBCT-based online ART. The memories of LSTM-UNet undergo updates at each fraction, and the model is sequentially trained and tested for each fraction in chronological order. Images are cropped for better illustration.

To overcome this limitation, we developed an enhanced 3D U-Net model embedded with LSTM modules, which we aptly named LSTM-UNet. This model excels in incorporating all available prior adaptive data to markedly enhance auto-segmentation quality on CBCT or sCT for the current fraction. The efficacy and framework of our LSTM-UNet are depicted in Fig. 1.

The model concatenates previous image and contours, and the current fraction's sCT into a 3-channel input. The prediction is the contours for the current sCT. It undergoes sequential training from the first to the last fraction. The model is specifically designed to use the pre-plan’s pCT+contours for the first fraction and, starting from the second fraction, the sCTs and their respective contours from subsequent adaptive fractions. Prior to the first fraction, the model's memory initializes to 0. With each new fraction included as input, LSTM-UNet can enhance its memory by assimilating more information about the current patient.

### 2.2 U-Net with LSTM

**Fig. 2:**
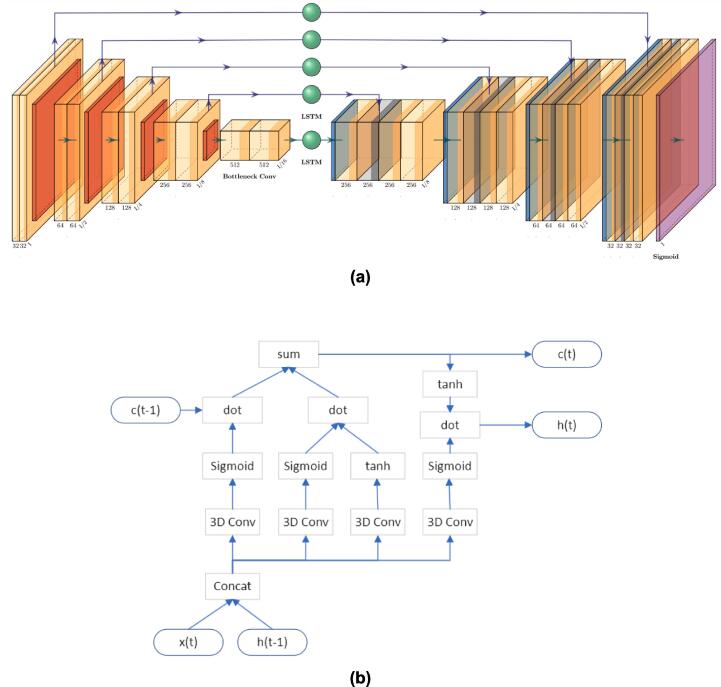
The proposed LSTM-UNet architecture. (a) The LSTM-UNet incorporates LSTM units inserted into each resolution of a standard 3D U-Net. (b) The structure of 3D LSTM units involves variables such as x(t) representing the current input, c(t-1) and h(t-1) representing the current state and hidden state from the preceding results, and c(t) and h(t) denoting the LSTM outputs of the current fraction.

The proposed workflow incorporates LSTM-UNet with architecture illustrated in Fig. 2. It constitutes a U-Net with 3D convolution [27], wherein an LSTM unit [28] is inserted during each skip connection phase. The structure of LSTM units is detailed in Fig. 2b. Given the input x(t), representing the features of the 3 channels’ input, along with the hidden state h(t-1) and current state $c\left(t-1\right)$ which includes information from all previous fractions, the model computes new h(t) and c(t) for the current fraction. These h(t) and c(t) states continually evolve with each input fraction, encapsulating the patient’s historical information. There are 5 features from the encoder and each feature is the input x(t) to the corresponding LSTM unit. The 5 outputs c(t) form the input to the decoder. The memories at different resolutions create a multi-level perception to track the anatomy change across the treatment course. The internal functions of LSTM units involve 3D convolutions and 3D activations. To manage the model size, the initial features are reduced to 32. During training, patient order is randomized per epoch to prevent overfitting. To align with Adaptive Radiation Therapy (ART) principles, we clear the LSTM memory before and after each patient, focusing the memory on the current patient's data. The batch size is set to 1 which accommodates GPU memory limitations. We have established a pre-training dataset and a clinical dataset, which will be discussed further. Our approach utilizes the RMSProp optimizer [29], and the loss function consists of binary cross-entropy and Dice loss with equal weights. The initial learning rate is set at $10^{-5}$. We used a gradient scheduler “ReduceLROnPlateau” and the learning rate is reduced automatically when loss is on plateau. We commence with 40 epochs for pre-training and follow up with 30 epochs for fine-tuning on the clinical dataset, which is just sufficient to reduce the learning rate to $10^{-8}$ and let the network converge. The last pre-trained checkpoint will be loaded for the fine-tuning.

### 2.3 Data

Because of the scarcity of available clinical data for ART, we simulated a multi-fraction dataset to pre-train our model. The dataset was simulated from 93 H&N patients, each of them having a pCT with manual contours and a CBCT. Using a DL-based DIR model [20], we calculated the deformation vector field (DVF) from the pCT to the CBCT and performed structure propagation to transfer contours from pCT to CBCT as the CBCT contours. Then we interpolated between -DVF and DVF to mimic anatomical changes in different fractions. In this way, we simulated 5 fractions for each patient with -DVF, −0.5 × DVF, 0, 0.5 × DVF, and DVF applied to both CBCT images and CBCT contours which helped the model to learn important CT image features. Besides simulation dataset, we also collected a real clinical dataset from Ethos ART system. It consists of 10 patients and each patient includes a pCT, manual contours on pCT, and 7 fractions. Each fraction has an sCT generated by Ethos system from the pCT, and the manual contours on sCT. The 10 patients from the Ethos dataset were divided into 4 for fine-tuning, 2 for validation, and 4 for testing. To avoid the misalignment of input images, rigid registration was performed between the images from the previous fraction and current fraction before their input into the model. The segmentation accuracy of 8 structures were evaluated using Dice similarity coefficient (DSC): left parotid (L Parotid), right parotid (R Parotid), left brachial plexus (L BPlex), right brachial plexus (R BPlex), esophagus, left submandibular gland (L SMG), right submandibular gland (R SMG), and nodal GTV (GTVn).

This study was approved and authorized by the internal review board.

## 3 Results

Hausdorff distance results can be found in the Supplemental Materials.

### 3.1 LSTM-UNet

Firstly, we compared the LSTM-UNet model to three baseline models, including: UNet-DS, which represents a segmentation model using only the sCT from the current fraction; Contour-prior, which represents revised contours from the previous fraction image; and UNet-prior, a U-Net-based segmentation model that incorporates prior information but lacks memory. The results are detailed in Table 1.

**Table 1:** The average Dice Similarity Coefficient (DSC) was calculated for predicted segmentations by various models across 4 testing patients, each with 7 fractions.

Structure	UNet-DS	Contour-prior	UNet-prior	LSTM-UNet
L Parotid	0.733 ± 0.078	0.854 ± 0.082	0.869 ± 0.073	**0.882 ± 0.070**
R Parotid	0.725 ± 0.099	0.871 ± 0.084	0.892 ± 0.065	**0.894 ± 0.073**
L BPlex	0.467 ± 0.099	0.625 ± 0.214	0.643 ± 0.183	**0.652 ± 0.182**
R BPlex	0.437 ± 0.151	0.687 ± 0.150	0.705 ± 0.128	**0.711 ± 0.143**
Esophagus	0.430 ± 0.119	0.734 ± 0.189	0.783 ± 0.120	**0.803 ± 0.115**
L SMG	0.627 ± 0.103	0.793 ± 0.092	0.809 ± 0.084	**0.816 ± 0.088**
R SMG	0.605 ± 0.113	0.781 ± 0.111	0.775 ± 0.115	**0.798 ± 0.106**
GTVn	0.170 ± 0.246	0.782 ± 0.105	0.787 ± 0.100	**0.795 ± 0.112**
Model size	99 MB	NA	198 MB	175 MB

Notably, UNet-DS failed to predict accurate segmentations. Utilizing contours from the previous fraction with rigid registration, Contour-prior served as a reasonable starting point. Hence, by incorporating image and contours from the previous fraction as additional input, both UNet-prior and LSTM-UNet notably improved segmentation accuracy compared to Contour-prior. However, UNet-prior is not as good as LSTM-UNet because it has no memories about past fractions. Comparing UNet-prior with LSTM-UNet, a paired sample *t*-test on the concatenated array of the 8 sample structures revealed a P-value = 0.0035, signifying a significant difference between UNet-prior and LSTM-UNet. LSTM-UNet demonstrated superior performance across each tested structure, attributed to its ability to retain memories from all previous fractions.

### 3.2 DIR+LSTM-UNet

To fully harness the potential of LSTM-UNet, we aimed to enhance the accuracy of the prior knowledge itself. As observed in Table 1, the performance of both the LSTM-UNet and UNet-prior models heavily relies on the accuracy of Contour-prior. For instance, UNet-DS shows similar accuracy on R BPlex and Esophagus (0.437 and 0.430, respectively). However, with a better Contour-prior accuracy for Esophagus (0.734–0.687), both UNet-prior and LSTM-UNet significantly improved their scores on Esophagus. Therefore, we opted to enhance the accuracy of the prior knowledge by replacing the contours from the previous fraction with deformed contours. Since the prior image should match prior contours, we used current image as the prior image, which means we have two input channels of the same current image. The Contour-prior segmentations were adjusted using a state-of-the-art deep learning-based Deformable Image Registration (DIR) method [21] between the images of the previous and current fractions, which is significantly better than Contour-prior. The LSTM-UNet model utilizing deformed registered Contour-prior as input is referred to as DIR+LSTM-UNet. A comparison among DIR-only derived Contour-prior (DIR), LSTM-UNet, and DIR+LSTM-UNet is presented in Table 2. A paired sample *t*-test between DIR and DIR+LSTM-UNet results on 8 sample structures revealed a P-value < 0.0001, signifying a significant difference between DIR segmentation and DIR+LSTM-UNet segmentation.

**Table 2:** The average Dice Similarity Coefficient (DSC) of the LSTM-UNet, DIR, and DIR+LSTM-UNet models across the same test set as Table 1.

Structure	LSTM-UNet	DIR	DIR+LSTM-UNet
L Parotid	0.882 ± 0.070	0.918 ± 0.028	**0.930 ± 0.027**
R Parotid	0.894 ± 0.073	0.930 ± 0.017	**0.942 ± 0.017**
L BPlex	0.652 ± 0.182	0.847 ± 0.043	**0.869 ± 0.035**
R BPlex	0.711 ± 0.143	0.866 ± 0.041	**0.887 ± 0.016**
Esophagus	0.803 ± 0.115	0.880 ± 0.045	**0.897 ± 0.034**
L SMG	0.816 ± 0.088	0.875 ± 0.033	**0.889 ± 0.030**
R SMG	0.798 ± 0.106	0.862 ± 0.051	**0.873 ± 0.053**
GTVn	0.795 ± 0.112	0.825 ± 0.058	**0.852 ± 0.044**

**Fig. 3:**
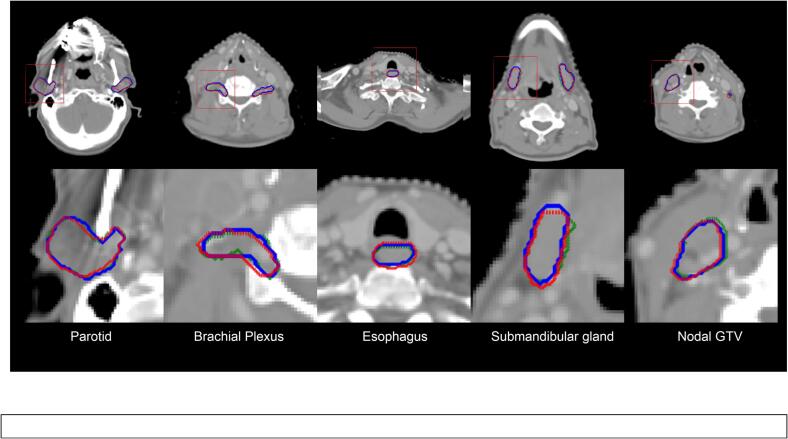
Samples of predicted segmentation by LSTM-UNet, DIR+LSTM-UNet, and ground truth. Green: Ground truth. Red: LSTM-UNet. Blue: DIR+LSTM-UNet. (For interpretation of the references to colour in this figure legend, the reader is referred to the web version of this article.)

It is important to note that certain individual slices demonstrated notably difference (as shown in Fig. 3) rather than an average improve on most single slices. The improved segmentation on such slices helps reduce the precious time for clinicians spent on revising the auto-generated contours and can potentially accelerate the clinical workflow of online ART.

**Fig. 4:**
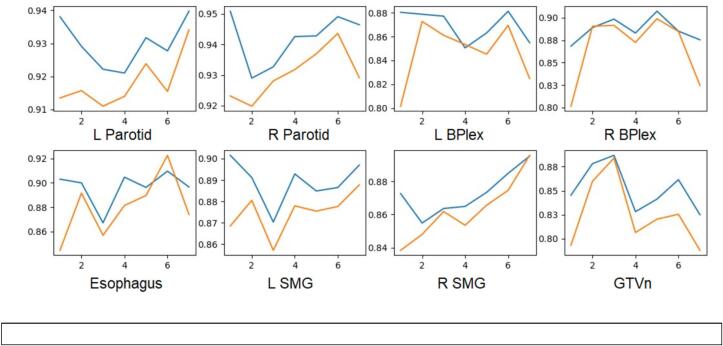
Fraction-Dice plot of DIR and DIR+LSTM-UNet of 8 structures. Orange: DIR. Blue: DIR+LSTM-UNet. Dice score of each curve is averaged among all the patients tested. (For interpretation of the references to colour in this figure legend, the reader is referred to the web version of this article.)

Fig. 4 illustrates that the DSC score of LSTM-UNet (blue) closely mirrors the DSC score of DIR (orange) across all fractions. Given that the LSTM-UNet is trained using contours from the previous fraction as one of its inputs, the segmentation prediction heavily relies on the accuracy of this input contour.

## 4 Discussion

Auto-segmentation of H&N organs and targets can be a difficult task. In this study, we introduced an auto-segmentation model tailored for CBCT-based online ART. This model can cumulate knowledge with each additional fraction to predict the segmentation of the next fraction. To do so we constructed a specialized 3D LSTM-UNet, which integrates LSTM memory modules to allow U-Net to retain learned information across previous fractions and enhance the segmentation precision of the current fraction. We conducted comprehensive experiments to benchmark the performance of our proposed model against other state-of-the-art segmentation models that either do not incorporate prior knowledge or lack an embedded memory mechanism. We also compared our model's efficacy with a state-of-the-art DIR-based segmentation method. Our proposed method outperforms models lacking prior knowledge or memory units and works together with DIR-derived segmentations. Compared to other groups’ auto-segmentation result on H&N CT or CBCT [18], our method has a higher accuracy on parotid gland (94 % compared to under 85 %), submandibular glands (88 % compared to 80 %) and GTV (85 % compared to 78 %). The improved auto-segmentation results help reduce the delay of contour revision and potentially accelerate the clinical workflow of online ART.

This work is currently based on sCT, which represents planning CT deformed to CBCT's anatomy. In clinical practice, sCT and its contours are ultimately used for dose calculation. Clinicians initially contour CBCT with influencers to protect critical organs, then generate the sCT image and contours based on CBCT. As the influencers are also transformed to sCT in previous fractions, the LSTM-UNet can learn and utilize these influencers as well. Moreover, this method can be directly implemented on CBCT segmentation with a few labeled data for finetuning. Similarly, this method can be implemented on other disease sites with corresponding CT and CBCT data for pre-training and fine-tuning.

Future advancements in prior-guided segmentation involve enhancing segmentation results from one fraction to the next. Presently, LSTM-UNet's prediction relies on the guidance of input prior knowledge. However, the model's predictive ability should improve progressively with the accumulation of more prior knowledge, leading to a nearly monotonically increasing Dice curve instead of the pattern observed in Fig. 4. This could be fixed by few-shot learning of the anatomical change between each fraction. Another limitation is we need more data to achieve statistical significance on every structure. Therefore, we plan to collect more data and perform multi-fold cross validation in a future study.

In summary, adaptive radiotherapy is a revolutionary paradigm in the field of radiation oncology, allowing for the patient’s change in anatomy to be reflected in their contours and plans. While current state-of-the-art segmentation models ignore the images and contours from previous fractions, our proposed LSTM-UNet analyzes both the current scan’s data and previous ground truth to derive an appropriate contour. The proposed progressive learning scheme and LSTM-UNet can reduce the burden of contouring for clinicians.

## Credit author statement

All the authors of this paper have written, informed consent for the publication of this paper.

## Declaration of competing interest

The authors declare that they have no known competing financial interests or personal relationships that could have appeared to influence the work reported in this paper.
