# Supplementary material for: Progressive auto-segmentation for cone-beam computed tomography-based online adaptive radiotherapy
Source: Phys Imaging Radiat Oncol. 2024 Jul 14;31:100610. doi: 10.1016/j.phro.2024.100610 (PMC11315102; doi:10.1016/j.phro.2024.100610)
Supplement: Supplementary Data 1 [file mmc1.docx]

**Supplemental materials**

**Table 1:**

95% Hausdorff 3D distance (3D HD95) evaluated on results of three models: prior contours, U-Net-64 without memories and LSTM-UNet. The unit is 1 mm. This table corresponds to Table 1 in the paper with only different metrics. For most of the structures, LSTM-UNet has the best 3D HD95 score, except for brachial plexuses which have poor registration.

| **Table 1.** 3D HD95 results of 3 models corresponding to Table 1 in the paper. The unit is 1mm. | | | |
| --- | --- | --- | --- |
| **Structure** | **Contour-prior** | **UNet-prior** | **LSTM-UNet** |
| L Parotid | 4.36 | 4.10 | 3.90 |
| R Parotid | 4.16 | 3.60 | 3.55 |
| L BPlex | 4.57 | 4.60 | 4.60 |
| R BPlex | 3.75 | 3.95 | 4.10 |
| Esophagus | 4.31 | 3.61 | 3.09 |
| L SMG | 4.09 | 4.00 | 3.91 |
| R SMG | 4.40 | 4.53 | 4.30 |
| GTVn | 5.38 | 5.41 | 5.35 |
| **Model size** | NA | 198MB | 175MB |

**Table 2:**

3D HD95 distance evaluated DIR input and DIR+LSTM-UNet prediction. The unit is 1mm. This table corresponds to Table 2 in the paper with only different metrics. DIR+LSTM-UNet has the best HD95 score over all the structures.

| **Table 2.** 3D HD95 results of 2 models corresponding to Table 2 in the paper. The unit is 1mm. | | |
| --- | --- | --- |
| **Structure** | **DIR** | **DIR+LSTM-UNet** |
| L Parotid | 2.68 | 2.55 |
| R Parotid | 2.41 | 2.28 |
| L BPlex | 2.10 | 2.05 |
| R BPlex | 2.08 | 2.00 |
| Esophagus | 2.13 | 2.03 |
| L SMG | 2.66 | 2.43 |
| R SMG | 2.98 | 2.85 |
| GTVn | 3.99 | 3.85 |

**Figure 1:**

Figure 1 illustrates the per-fraction dice curve of the results in Table 1 in the paper. From the plot we can see LSTM-UNet is better than UNet-prior in most of the fractions and structures except for the second fraction of left and right brachial plexus. This is because the prior knowledge of brachial plexus is poorly registered in the second and third fraction. This plot again signifies the importance of high-quality prior knowledge.

| 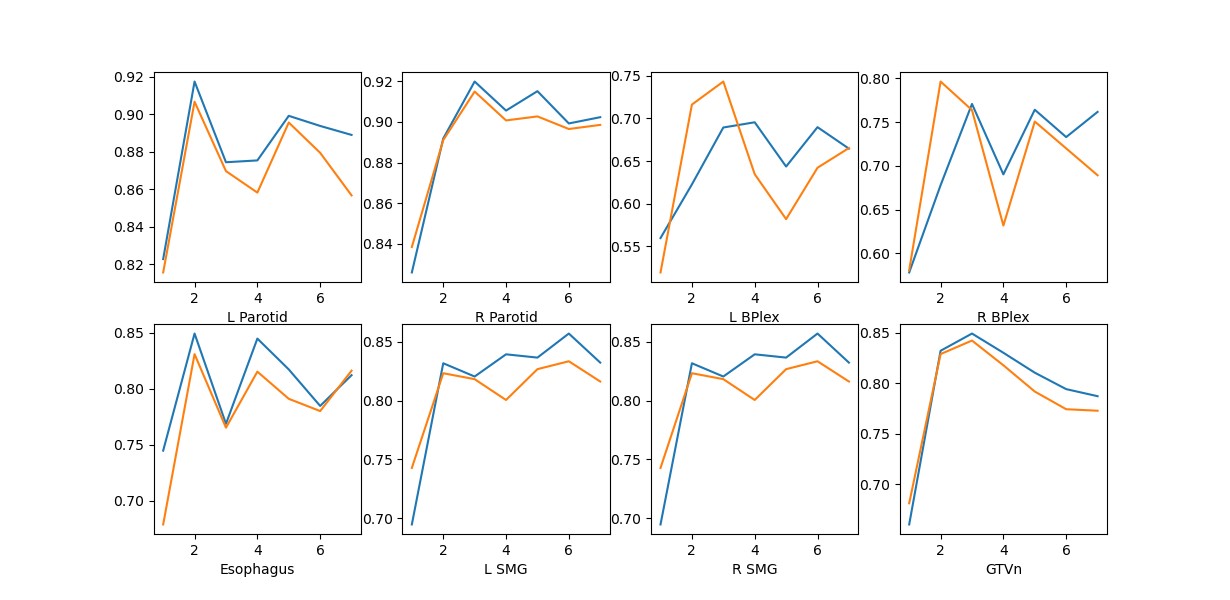 |
| --- |
| **Figure 1**. Fraction-Dice plot of UNet-prior and LSTM-UNet of 8 structures. Orange: UNet-prior. Blue: LSTM-UNet. Dice score of each curve is averaged among all the patients tested. |
